# Outcomes of intraventricular 131-I-omburtamab and external beam radiotherapy in patients with recurrent medulloblastoma and ependymoma

**DOI:** 10.1007/s11060-022-04235-w

**Published:** 2023-02-28

**Authors:** Kathryn R. Tringale, Suzanne L. Wolden, Matthias Karajannis, Sofia Haque, Luca Pasquini, Onur Yildirim, Marc Rosenblum, Jamal K. Benhamida, Nai-Kong Cheung, Mark Souweidane, Ellen M. Basu, Neeta Pandit-Taskar, Pat B. Zanzonico, John L. Humm, Kim Kramer

**Affiliations:** 1 Department of Radiation Oncology, Memorial Sloan Kettering Cancer Center, New York, NY USA; 2 Department of Pediatrics, Memorial Sloan Kettering Cancer Center, New York, NY USA; 3 Department of Radiology, Memorial Sloan Kettering Cancer Center, New York, NY USA; 4 Department of Pathology, Memorial Sloan Kettering Cancer Center, New York, NY USA; 5 Department of Neurosurgery, Memorial Sloan Kettering Cancer Center, New York, NY USA; 6 Department of Nuclear Medicine, Memorial Sloan Kettering Cancer Center, New York, NY USA; 7 Department of Medical Physics, Memorial Sloan Kettering Cancer Center, New York, NY USA

**Keywords:** Intraventricular compartmental radioimmunotherapy, External beam radiotherapy, Proton beam radiotherapy, Pediatric brain tumors, Radiation necrosis

## Abstract

**Purpose:**

Intraventricular compartmental radioimmunotherapy (cRIT) with 131-I-omburtamab is a potential therapy for recurrent primary brain tumors that can seed the thecal space. These patients often previously received external beam radiotherapy (EBRT) to a portion or full craniospinal axis (CSI) as part of upfront therapy. Little is known regarding outcomes after re-irradiation as part of multimodality therapy including cRIT. This study evaluates predictors of response, patterns of failure, and radiologic events after cRIT.

**Methods:**

Patients with recurrent medulloblastoma or ependymoma who received 131-I-omburtamab on a prospective clinical trial were included. Extent of disease at cRIT initiation (no evidence of disease [NED] vs measurable disease [MD]) was assessed as associated with progression-free (PFS) and overall survival (OS) by Kaplan–Meier analysis.

**Results:**

All 27 patients (20 medulloblastoma, 7 ependymoma) had EBRT preceding cRIT: most (22, 81%) included CSI (median dose 2340 cGy, boost to 5400 cGy). Twelve (44%) also received EBRT at relapse as bridging to cRIT. There were no cases of radionecrosis. At cRIT initiation, 11 (55%) medulloblastoma and 3 (43%) ependymoma patients were NED, associated with improved PFS (p = 0.002) and OS (p = 0.048) in medulloblastoma. Most relapses were multifocal. With medium follow-up of 3.0 years (95% confidence interval, 1.8–7.4), 6 patients remain alive with NED.

**Conclusion:**

For patients with medulloblastoma, remission at time of cRIT was associated with significantly improved survival outcomes. Relapses are often multifocal, particularly in the setting of measurable disease at cRIT initiation. EBRT is a promising tool to achieve NED status at cRIT initiation, with no cases of radiation necrosis.

**Supplementary Information:**

The online version contains supplementary material available at 10.1007/s11060-022-04235-w

## Introduction

Curative treatment options for patients with recurrent primary tumors of the central nervous system (CNS) are limited. Many patients have had previous multimodality therapy, including external beam radiotherapy (EBRT), which introduces the potential for toxicity with additional treatments. For instance, radiation necrosis is a potential toxicity with an incidence of up to 5% [1, 2]. Intrathecal compartmental radioimmunotherapy (cRIT) using radio-iodinated 131-I-3F8 or 131-I-omburtamab offers another therapeutic strategy for CNS malignancies by eradicating cancer cells in the cerebrospinal fluid (CSF) without long-term neurologic toxicity [3, 4]. Omburtamab targets tumor antigen B7-H3, a cell surface protein found on most solid tumors. By injecting radio-labeled omburtamab into the CSF using an Ommaya catheter, 131-I-omburtamab has access to tumor cells in the CNS compartment with a highly favorable therapeutic index between CSF and blood [5]. Improvement in survival has been found using 131-I-omburtamab in relapsed CNS neuroblastoma [3].

For medulloblastoma, 131-I-3F8 has been shown to be safe and may have clinical utility in maintaining remission in high-risk or recurrent disease [6]. Prior data investigating cRIT in combination with EBRT showed minimal risk (~ 1%) of radionecrosis in children with metastatic neuroblastoma and medulloblastoma [7]. Predictors of response, patterns of failure, and radiologic events following 131-I-omburtamab in patients with primary CNS malignancies, particularly those who also receive EBRT, are not clear. Since EBRT is standard treatment in the upfront setting for medulloblastoma and ependymoma, the clinical scenario of considering re-irradiation at relapse, particularly in anticipation of cRIT, is challenging. Prior studies have focused on the incidence of radiation necrosis but have yet to evaluate other potential radiologic findings (i.e., cavernomas) in these heavily treated patients. We sought to assess clinical outcomes and radiologic events in patients with recurrent medulloblastoma and ependymoma who received cRIT to understand the role of bridging therapy.

## Materials and methods

### Eligible patients

Patients with recurrent medulloblastoma or ependymoma known to express B7H3 by immunohistochemical staining were eligible to receive cRIT with 131-I-omburtamab at Memorial Sloan Kettering Cancer Center (MSK) on an IRB-approved phase I protocol (NCT# 00089245) from 2004 TO 2019. Patients were eligible for the protocol if they had no rapidly deteriorating neurologic examination or obstructive hydrocephalus, an absolute neutrophil count > 1000/ul, platelet count > 50,000/ul, serum bilirubin < 3.0 mg/dl, and serum creatinine < 2 mg/dl. Ommaya catheter position, patency, and CSF flow were evaluated in all patients before treatment using 111-Indium diethylene triamine pentaacetic acid scintigraphy studies. There was no prior EBRT dose limit and at least a 3-week interval between most recent EBRT and cRIT. Patients and/or guardians provided informed consent for trial enrollment. For the purposes of evaluating patients with recurrent primary brain tumors, patients with recurrent ependymoma or medulloblastoma who received cRIT and at least one imaging response assessment were included.

### 131-I-omburtamab administration

cRIT with 131-I-Omburtamab was delivered as previously described: a test dose (2 milliCurie [mCi]/injection), then if tolerated, followed by 1 or 2 monthly injections (10-70 mCi/injection) with dosing based on a phase I dose-escalation level at the time of patient entry. Clinical status, vital signs and neurologic examination were monitored overnight. Repeat therapy injections were administered in the absence of grade three or four toxicity [8].

### Bridging therapies

Bridging therapy was defined as treatment delivered after diagnosis of the most recent relapse prior to initiation of cRIT. Specifically, surgical resection, EBRT (focal and/or CSI), and medical therapy (both systemic and intrathecal chemo- or immunotherapies) at time of most recent relapse prior cRIT were noted. In addition to bridging therapies, treatment details of all EBRT (e.g., receipt of CSI, delivery with protons, dose, and number of treatments) both pre- and post-cRIT were collected.

### Evaluation of response

All patients had conventional MRI of the brain and spine within 3 weeks prior to cRIT and approximately 5 weeks after each therapy administration along with CSF collection. MRIs included sagittal and axial T1-weighted, axial T2-weighted, axial fluid-attenuated inversion recovery (FLAIR), axial diffusion weighted images, apparent diffusion coefficient and exponential maps, and postcontrast T1-weighted sequences in axial, sagittal, and coronal planes [9]. Patients with disease seen on contrast-enhanced MRI of the brain or spine, or with malignant cells in the CSF cytology obtained by ventricular sampling, were considered to have measurable disease (MD) at cRIT initiation. Patients who had a complete response to bridging therapy prior to cRIT were considered to have no evidence of disease (NED) at baseline. After the initial 5-week post-cRIT MRIs as described, patients underwent additional MRIs approximately every 3 months for the first year, and every 6–12 months annually thereafter.

Initial response on the first post-cRIT MRI and later recurrence on future MRIs were assessed by three board-certified radiologists per RANO/RANO-LM guidelines [10–12]. A complete response (CR) was defined as total regression of disease and absence of new disease on MRI and/or clearance of CSF cytology. Partial response (PR) was defined by ≥ 50% decrease in tumor size or decrease in leptomeningeal enhancement. Stable disease was defined as < 50% decrease or no reduction in tumor size demonstrable by MRI, or tumor growth that is less than the criteria defined as progressive disease. Progressive disease was defined as new disease or clinical or radiological evidence of increased volume > 25% in tumor area with maximum perpendicular diameter in any site of residual disease compared to immediate pre-study area or increased leptomeningeal enhancement on MRI. CSF cytology was also evaluated.

Patterns of relapse (e.g., intraparenchymal and/or spine, focal or multifocal/diffuse, CSF cytology) were evaluated. Radiologic events (e.g., radionecrosis, cavernomas) were assessed.

### Statistics

Progression-free survival (PFS) was calculated both from time of first 131-I-omburtamab injection and from time of relapse pre-cRIT to progression, relapse, or death. Overall survival (OS) was calculated from time of first 131-I-omburtamab injection and time of pre-cRIT relapse until death. Extent of disease at cRIT initiation (no evidence of radiologic and cytologic disease [NED] v measurable disease [MD]) along with bridging therapies were evaluated as predictors of PFS and OS by Kaplan–Meier analysis with log-rank tests. Median follow-up was calculated using reverse Kaplan–Meier analysis with death as a censoring event. Time-to-event analyses were stratified by primary histology. Two-sided *P-*values < 0.05 were considered significant. Analyses were performed with SAS (SAS Enterprise Guide, version 7.1).

## Results

### Baseline characteristics

Twenty-seven patients (20 medulloblastoma, 7 ependymoma) enrolled on the protocol who underwent imaging response assessment were included with a median follow-up of 3.0 years (95% confidence interval [CI], 1.8–7.4; Supplemental Fig. 1). Of the 13 medulloblastoma patients with molecular subtyping, most (9, 45%) were non-WNT/non-SHH. One patient with medulloblastoma had underlying Li-Fraumeni Syndrome with a pathogenic germline p53 mutation (Table 1). Median age at cRIT initiation was 12 years (range, 3–40).

**Table 1:** Baseline patient and disease characteristics

	**Medulloblastoma**		**Ependymoma**	
N (%)	20 (74)		7 (26)	
**Age **(median, range) At diagnosis At first dose cRIT At first EBRT course	9 (4–33) 13 (6–40) 9 (4–33)		4 (1–12) 9 (3–13) 4 (2–12)	
**Gender**				
Male Female	12 (60) 8 (40)		5 (71) 2 (29)	
**M Stage at Diagnosis** M0 M1 M2 M3 Unknown	15 (75) 0 0 4 (20) 1 (5)		7 (100) 0 0 0 0	
**Extent of Initial Surgery** STR GTR	4 (20) 16 (80)		2 (29) 5 (71)	
**Disease Characteristics**				
	**Histology** Nodular, desmoplastic Classic Large cell/anaplastic Unknown or unclear	2 (10) 10 (50) 4 (20) 4 (20)	**WHO Grade ** II III	2 (29) 5 ( 71)
	**Molecular Subtype** SHH-activated Non-WNT/non-SHH Unknown	4 (20) 9 (45) 7 (35)	**Tumor Location at Dx** Supratentorial Infratentorial (posterior fossa	1 (14) 6 (86)
	**Risk Stratification** Average High	15 (75) 5 (25)		

2^*^1 presumed SHH-activated given Li Fraumeni Syndrome with p53 germline mutation

*cRIT* compartmental radioimmunotherapy; *EBRT* external beam radiotherapy; *STR* subtotal resection; *GTR* gross total resection

At cRIT initiation, 9 (45%) medulloblastoma and 4 (57%) ependymoma patients had radiologically MD (Table 2). One patient with medulloblastoma was radiologically NED but had malignant cells in the CSF and therefore was categorized as having MD. Median number of cRIT injections was 3 (range, 1–4) with median total dose of 72 mCi (range, 2–192). Two patients (both with medulloblastoma) only received a single test dose (2 mCi). Eight patients (30%) had previously received cRIT with 131-I-3F8 before receiving cRIT with 131-I-omburtamab.

**Table 2:** Disease Status at I-131-Omburtamab cRIT Initiation

	Total N = 27	Medulloblastoma 20	Ependymoma 7
Site of disease			
Brain	4 (15)	3 (15)	1 (17)
Spine	3 (11)	2 (10)	1 (17)
Both	4 (15)	3 (15)	2 (29)
Radiologically NED	16 (59)	11 (55)*	3 (50)
CSF status			
Positive	4 (15)	4 (20)	0 (0)
Negative	23 (85)	16 (80)	7 (100)
Bridging treatment to cRIT			
Local Therapy	20 (74)	16 (80)	6 (86)
Surgery	8 (40)	4 (25)	6 (100)
EBRT	4 (20)	4 (25)	0 (0)
Focal photons	2	2	0
Focal protons	1	1	0
CSI protons	1	1	0
Surgery + EBRT	8 (30)	8 (50)	0 (0)
Focal photons	7	7	0
CSI protons	1	1	0
Systemic and/or other IT therapy	4 (15)	3 (15)	1 (14)
None	3 (11)	1 (5)	0 (0)

Number (%)

*EBRT* external beam radiotherapy; *NED* no evidence of disease; *CSF* cerebrospinal fluid; *IT* intrathecal; *CSI* craniospinal irradiation

*One patient with medulloblastoma was radiologically NED but had malignant cells in the CSF and therefore was categorized as having MD

### External beam radiotherapy prior to 131-I-omburtamab

All patients had EBRT at a median time of 0.9 years (range 0.1–5.2) preceding cRIT (Supplemental Table 1). Most patients received CSI (22, 81%), with a median dose of 2340 cGy (range 2300–3960 cGy) with a boost to 5400 cGy (range 5040–6000 cGy). Ten (37%) CSI treatments were delivered with protons, all for patients with medulloblastoma. Most patients (20, 74%) received a focal course of EBRT, five of which were delivered with protons. Among patients with medulloblastoma, the maximum dose of EBRT pre-cRIT ranged from 1800 to 5000 cGy (median 3000 cGy). For ependymoma, the maximum dose of EBRT pre-cRIT ranged from 4720 to 5940 cGy (median 5700 cGy). Prior to cRIT, 15 (56%) medulloblastoma and 6 ependymoma (86%) patients received more than one course of EBRT. None of the patients received multiple courses of CSI pre-cRIT.

### Disease response

#### Medulloblastoma

**Fig. 1:**
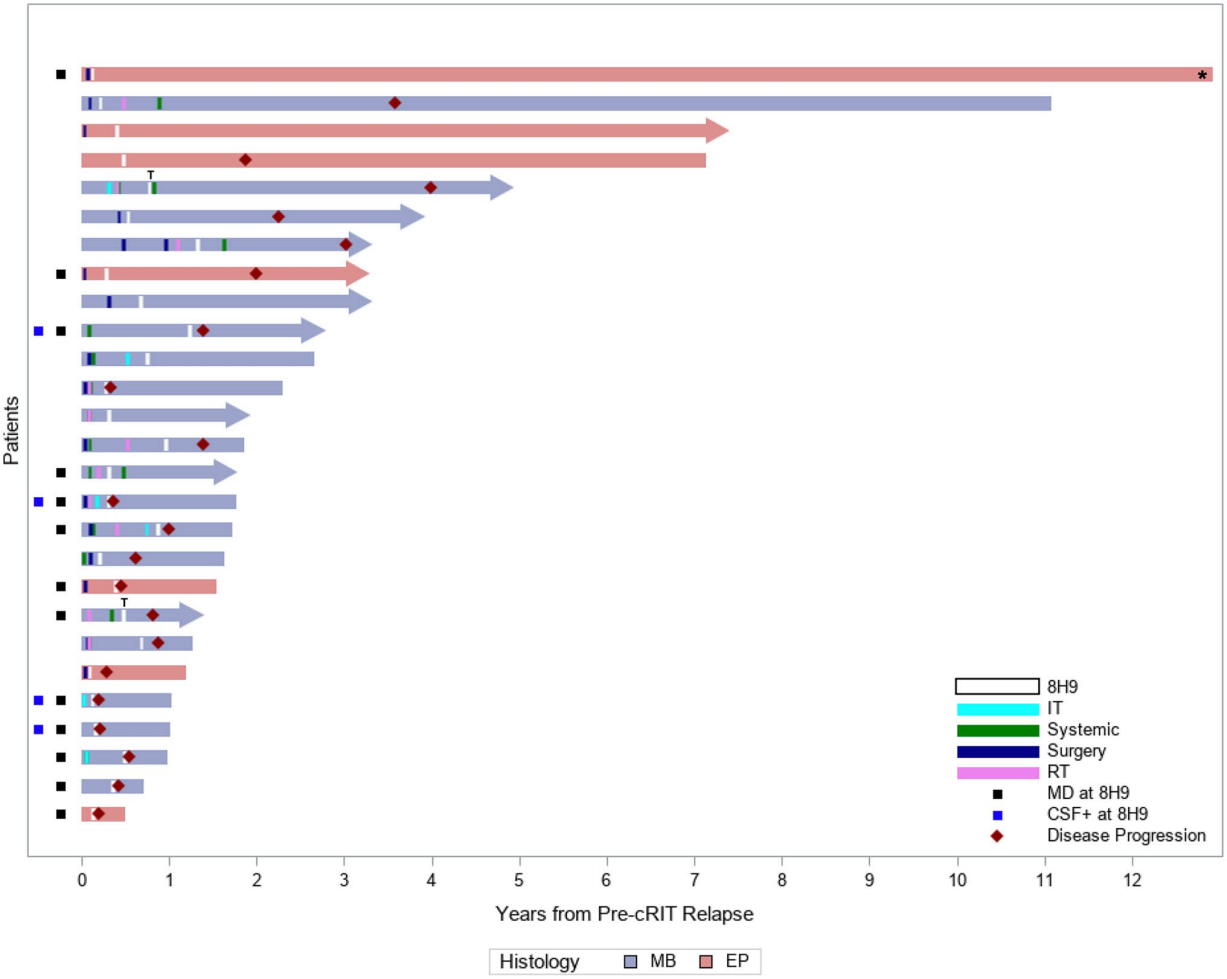
Swimmer’s plot demonstrating timing of treatments received and progression events. An arrowhead indicates that the patient was alive at the time of last follow-up. Those without an arrowhead indicate that the patient died at that time. *One patient was lost to follow-up and died of unknown causes. *MB* medulloblastoma; *EP* ependymoma; *MD* measurable disease; *CSF* cerebrospinal fluid; 8H9, I-131-omburtamab; cRIT compartmental radioimmunotherapy. ^T^Two patients (both with medulloblastoma) only received a single test dose (2 mCi)

All 20 patients received EBRT prior to cRIT at a median time of 0.6 years (range, 0.1–4.9). Most patients (12, 60%) received EBRT as part of bridging therapy, nearly all (11, 92%) of which were re-irradiation and three were with protons (2 CSI, 1 focal; Table 2). Surgical resection was commonly (12, 60%) used as part of bridging to cRIT. One patient received 131-I-3F8 as part of the bridging regimen to 131-I-omburtamab. Only three patients received additional systemic therapy after cRIT initiation. Nine patients had MD at cRIT initiation: multifocal radiologic disease in eight and isolated cytologic disease in one. Of the patients with baseline MD, five progressed on initial assessment, two had stable MD, and two achieved CR to cRIT. Of the two complete responders, one recurred 4 months after cRIT but is still alive and the other remained in remission 1.5 years after cRIT (Fig. 1).

Four patients remain in remission: at cRIT initiation, three had NED and one had multifocal leptomeningeal disease in the spine. Sixteen patients ultimately had disease progression at a median time of 0.8 years (range, 0.2–4.0) after most recent relapse and 0.2 years (range, 0.1–3.4) after cRIT initiation. Half of those who progressed had NED at cRIT initiation: four progressed focally intracranially, two progressed multifocally intracranially, and one had LMD in the spine per outside report (Supplemental Fig. 2). The other half who progressed had MD at cRIT initiation, and these progression patterns were nearly all multifocal and/or diffuse with LMD, with the exception of one patient with isolated cytologic disease at baseline who ultimately developed focal intracranial LMD.

**Fig. 2:**
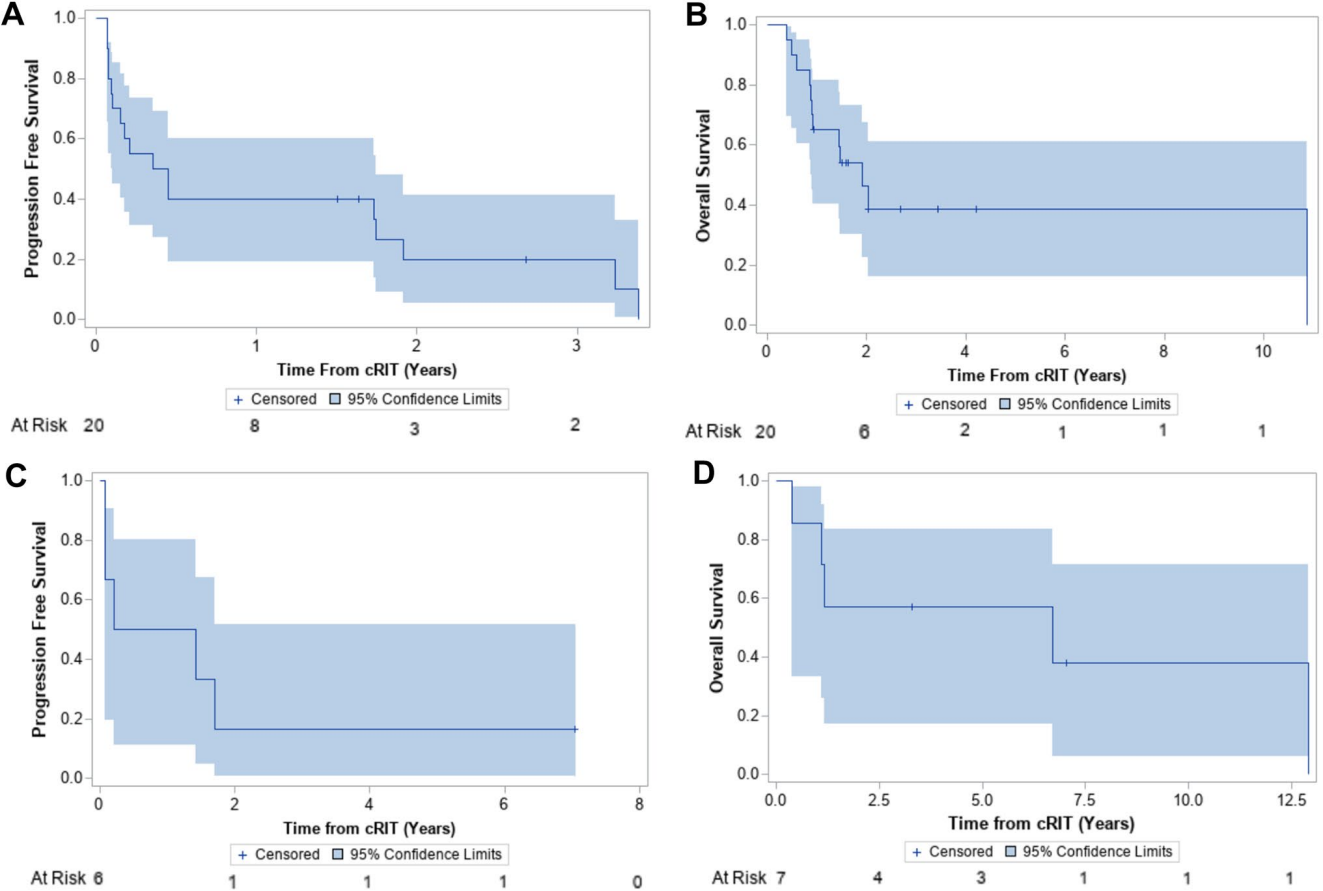
Survival outcomes among patients with medulloblastoma (top) and ependymoma (bottom) calculated from the time of pre-cRIT relapse. **A** Progression-free and **B** overall survival among patients with medulloblastoma. **C** Progression-free and **D** overall survival among patients with ependymoma. Note: one patient with ependymoma was lost to follow-up and was therefore not included in the progression free survival calculation

**Fig. 3:**
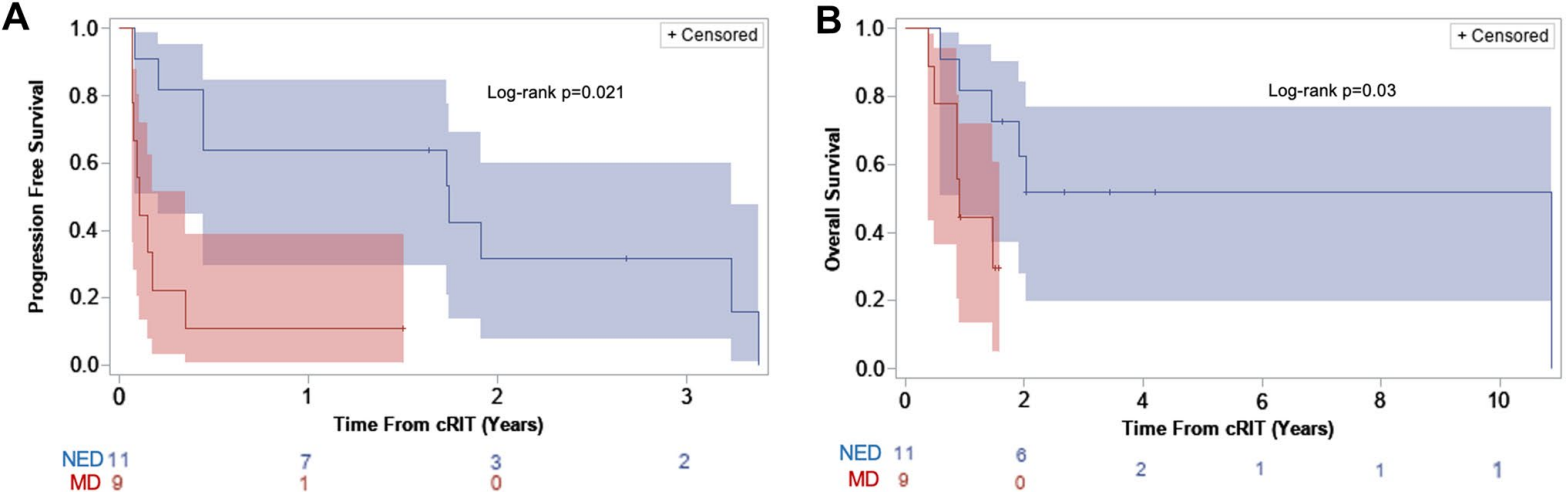
Survival outcomes among patients with medulloblastoma based on disease status at cRIT initiation. **A** Progression free and **B** overall survival from cRIT administration among patients with medulloblastoma. cRIT, compartmental radioimmunotherapy

Median PFS following cRIT for the medulloblastoma cohort was 0.4 years from cRIT initiation (95% CI 0.1–1.7; Fig. 2A) and 1.2 years from the most recent relapse preceding cRIT (95% CI 0.4–2.6). Patients who were NED at cRIT initiation had significantly improved PFS compared to those with MD (1.7 vs 0.1 years, p = 0.002; Fig. 3A). Median OS was 1.9 years after cRIT initiation (95% CI 0.9–10.9; Fig. 2B) and 2.3 years from most recent relapse pre-cRIT (95% CI 1.2–11.1). OS was improved among patients who were NED vs those who had MD at cRIT initiation (p = 0.048, Fig. 3B). The median PFS was numerically lower among non-SHH/WNT patients than SHH-activated patients (0.21 years [95% CI 0.07–3.24] vs 1.74 years [95%CI 1.73–1.92]; log-rank p = 0.23; Supplemental Fig. 3A). Similarly, the OS was also numerically lower among the non-SHH/WNT patients (0.44 years [95%CI 0.07-NR] vs 1.91 years [95%CI NR-NR]; log-rank p = 0.020; Supplemental Fig. 3B).

Among medulloblastoma patients, three underwent additional focal EBRT following cRIT for future recurrences. One patient received partial brain EBRT (2000 cGy), one patient received focal EBRT to the spine (2000 cGy), and one patient received three additional focal EBRT courses to the brain (maximum dose 3000 cGy). No patients received CSI post-cRIT.

#### Ependymoma

While all the patients with ependymoma had received EBRT at a median time of 1.2 years (range, 0.2–5.2) preceding cRIT, none had received EBRT as a bridge to cRIT after the most recent relapse (Table 2). All patients underwent surgical resection as a bridge to cRIT. Four patients had MD at cRIT initiation: two had LMD (one spinal alone, one both intracranial and spinal) and one with parenchymal disease. None had evidence of CSF involvement at cRIT initiation. No patients received additional systemic therapy after cRIT initiation.

Five patients ultimately had progression at a median time of 0.4 years (range, 0.2–1.9) after most recent relapse and 0.2 years (range, 0.1–1.4) after cRIT initiation: two who were NED at cRIT initiation ultimately relapsed at or adjacent to the site of initial intraparenchymal disease, one had progression of the pre-cRIT measurable LMD disease in the spine but had PR in the intracranial LMD, one failed both at the measurable pre-cRIT site in the brain as well as developed new LMD in the spine, and one failed in both the brain parenchymal and had LMD in the spine (Supplemental Fig. 1). One patient who had measurable spine LMD at cRIT initiation was lost to follow up after initial post-cRIT MRI showed stable disease and he ultimately died 12.8 years after cRIT initiation (Fig. 1). Median PFS (excluding the patient lost to follow-up who died of unknown causes) was 1.2 years from relapse (95% CI 0.2-NR) and 0.8 years from cRIT initiation (95% CI 0.1-NR; Fig. 2C).

Three patients underwent additional focal EBRT to the brain following cRIT for progression (one patient received 5940 cGy) or recurrence (one patient received 4 additional courses to multiple sites in the brain with a maximum dose of 5400 cGy, another patient underwent stereotactic radiosurgery of unknown total dose). No patients received CSI post-cRIT. The one patient who is alive at last follow-up without evidence of disease was originally NED at cRIT initiation. Median OS was 7.1 years from pre-cRIT relapse (95% CI 0.5–12.9) and 6.7 years from cRIT initiation (95% CI 0.4–12.9; Fig. 2D).

### Radiologic events

Of the 13 patients who had radiologic findings following cRIT, there were no cases of radionecrosis (Supplemental Table 2). The most common event identified was cavernoma (6, 46%), although all were asymptomatic and none required intervention. Three patients (23%) had hydrocephalus, one of whom was symptomatic secondary to acute cerebral aqueduct stenosis requiring shunt placement. One patient did ultimately develop posterior reversible encephalopathy syndrome (PRES) in the setting of pre-existing infarcts. However, this clinic-radiographic syndrome was diagnosed after the developing acute myeloid leukemia (AML). One patient had had ventricular prominence, one had subdural effusions, and one had intramedullary hemorrhage.

## Discussion

Patients with recurrent primary CNS tumors unfortunately have few curative treatment options. Here, we demonstrate that long-term survival over two years is possible among patients with recurrent medulloblastoma or ependymoma treated with multimodality therapy at relapse. These patients comprise a heavily treated population at risk of toxicity. With no cases of radionecrosis in this cohort despite all patients having at least one EBRT course pre-cRIT, our findings were consistent with previously published safety data [5, 7]. In fact, patients who had achieved remission at time of cRIT, often with aid of EBRT, had improved survival outcomes. Disease status at time of cRIT initiation is therefore likely critical, with patterns of failure demonstrating the multifocal nature of relapse, particularly in the setting of multifocal, measurable radiologic disease at cRIT initiation.

Patients who had NED at 131-I-omburtamab cRIT initiation had improved survival outcomes. This finding parallels previously published data for medulloblastoma patients who received 131-I-3F8 showing patients treated with cRIT while in remission had a lower risk of death compared to patients who had MD [6]. Eight patients with medulloblastoma lived more than 2 years after pre-cRIT relapse, all but one of whom were NED at cRIT initiation. Most patients who are still alive are in remission, one of whom had recurred following cRIT and was successfully salvaged. Approach to bridging therapy was heterogenous even within each approach (e.g., EBRT dosing and fractionation, spectrum of systemic therapies), which likely explains why bridging therapy approach was not an independent predictor of survival. Interestingly, only three patients received additional systemic therapy after cRIT. Therefore, while the method of bridging was not significant, achieving an NED status via bridging therapy to cRIT was an important predictor of survival among medulloblastoma. This clinical importance is particularly relevant in the context of recent data showing the safety of incorporating cRIT into multimodality therapies [5].

Disease status at cRIT initiation was significantly associated with PFS for medulloblastoma. Among the patients with medulloblastoma who died, nearly all died of progression or relapse, with one patient ultimately dying of treatment-related AML. Similarly, among ependymoma patients, all but one patient lost to follow-up were confirmed to have died from disease progression, highlighting the importance of achieving disease control for survival. When patients who were NED at cRIT initiation did ultimately fail, the patterns of failure tended to be more unifocal and intracranial, whereas patients with multifocal failures typically had multifocal, measurable disease at the time of cRIT. This phenomenon of achieving remission prior to cRIT parallels the consistent theme in cancer treatment to achieve CR or minimal residual disease to optimize outcomes of consolidative therapies, such autologous stem cell transplant [13, 14] and cellular therapies [15]. These data support multimodality bridging therapy to optimize patients’ disease status prior to receiving cRIT.

Despite all patients having received at least one course of EBRT prior to cRIT, 6 patients having received additional EBRT post-cRIT, and 8 patients having previously received cRIT with I-131-3F8, there was no evidence of radiation necrosis on post-cRIT MRIs. Our findings support prior safety data investigating cRIT in combination with EBRT [5, 7]. While this cohort study here is limited by sample size, this finding is particularly encouraging in the context of the uncertainty around radiation necrosis and brainstem injury risk in pediatric patients receiving EBRT with protons [16]. While some studies have cited higher rates of brainstem injury after EBRT with protons (up to 16.0%) as opposed to photons (up to 8.6%) [17], a recent National Cancer Institute workshop analysis of pediatric patients treated with focal proton therapy alone demonstrated a lower average rate of brainstem toxicity (2.4% symptomatic, 0.4% fatal) [18]. Our data further support a low risk of proton-related injury even in a heavily treated population of pediatric patients who received cRIT in addition to proton EBRT to the entire neuroaxis (CSI in 10 patients) or focally (in 6 patients). Moreover, several patients had proton therapy as part of their bridging regimen to cRIT (proton CSI in 2, focal proton therapy in 1), so while the sample size is small, we are encouraged by these findings. We also looked for other potential radiologic evidence of treatment effect beyond radiation necrosis, including cavernomas and hydrocephalus. Only patient had a symptomatic finding possibly related to therapy, and this hydrocephalus was reversed with placement of a VP shunt. Investigation is warranted to interpret the clinical relevance of these findings, as the majority were incidentally identified.

This study has several limitations. First, this cohort is heterogeneous with different histologies, therapies, and prognoses. Second, while these data on patients who received I-131-omburtamab were prospectively collected, delivery of EBRT and other treatments varied depending on the treating institution. Third, given patients received therapy at different institutions throughout their disease course, there is a possibility of missing records. Fourth, certain treatments included in this analysis are not widely available and may impact generalizability.

In conclusion, for patients with recurrent medulloblastoma or ependyma receiving cRIT, disease status at cRIT initiation may be critical. Among patients with medulloblastoma, radiologic and cytologic remission at 131-I-omburtamab initiation was associated with significantly improved survival. Patterns of relapse also demonstrated that patients with measurable disease at cRIT initiation were likely to fail multifocally and diffusely. For patients with the goal of cure, these findings support efforts to achieve remission prior to cRIT initiation using local bridging therapy (e.g., re-irradiation) as these patients can have improved survival outcomes.

## Supplementary Information

Below is the link to the electronic supplementary material.

Supplementary file1 (DOCX 766 KB)

## Data Availability

The datasets generated and/or analyzed during the current study are available from the corresponding author on reasonable request.
