# Supplementary material for: Outcomes of intraventricular 131-I-omburtamab and external beam radiotherapy in patients with recurrent medulloblastoma and ependymoma
Source: J Neurooncol. 2023 Feb 28;162(1):69–78. doi: 10.1007/s11060-022-04235-w (PMC10050019; doi:10.1007/s11060-022-04235-w)
Supplement: Supplementary file 1 — Supplementary file1 (DOCX 766 KB) [file 11060_2022_4235_MOESM1_ESM.docx]

**SUPPLEMENTAL**

**
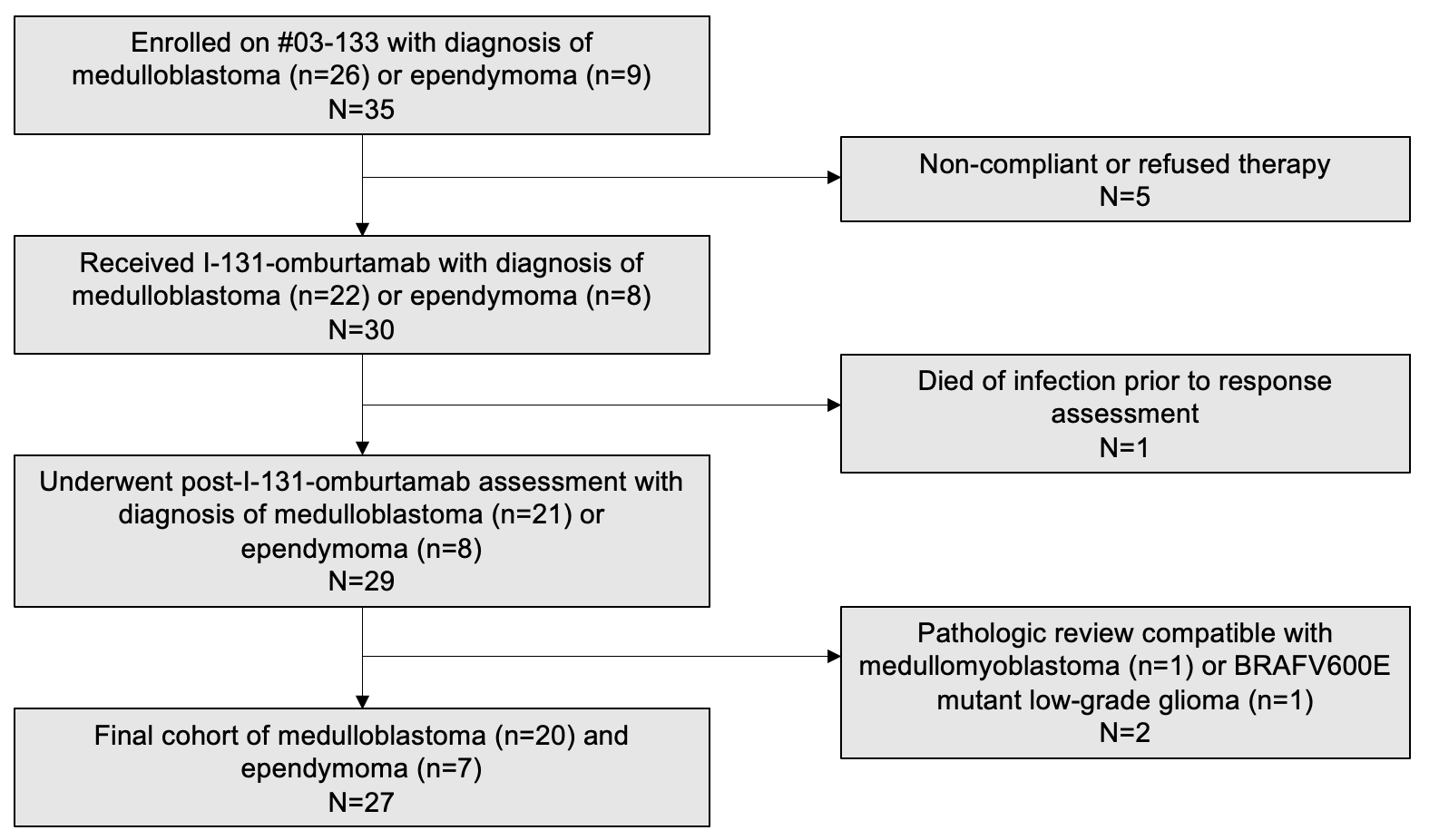
**

**Supplemental Figure 1.** Consort diagram outlining the patients who were included in this analysis. For the purposes of evaluating patients with recurrent primary brain tumors, patients with recurrent ependymoma or medulloblastoma who received cRIT and at least one imaging response assessment were included. One patient died of infection two months after receipt of I-131-ombutamab and the event was considered unrelated to the study drug.

**
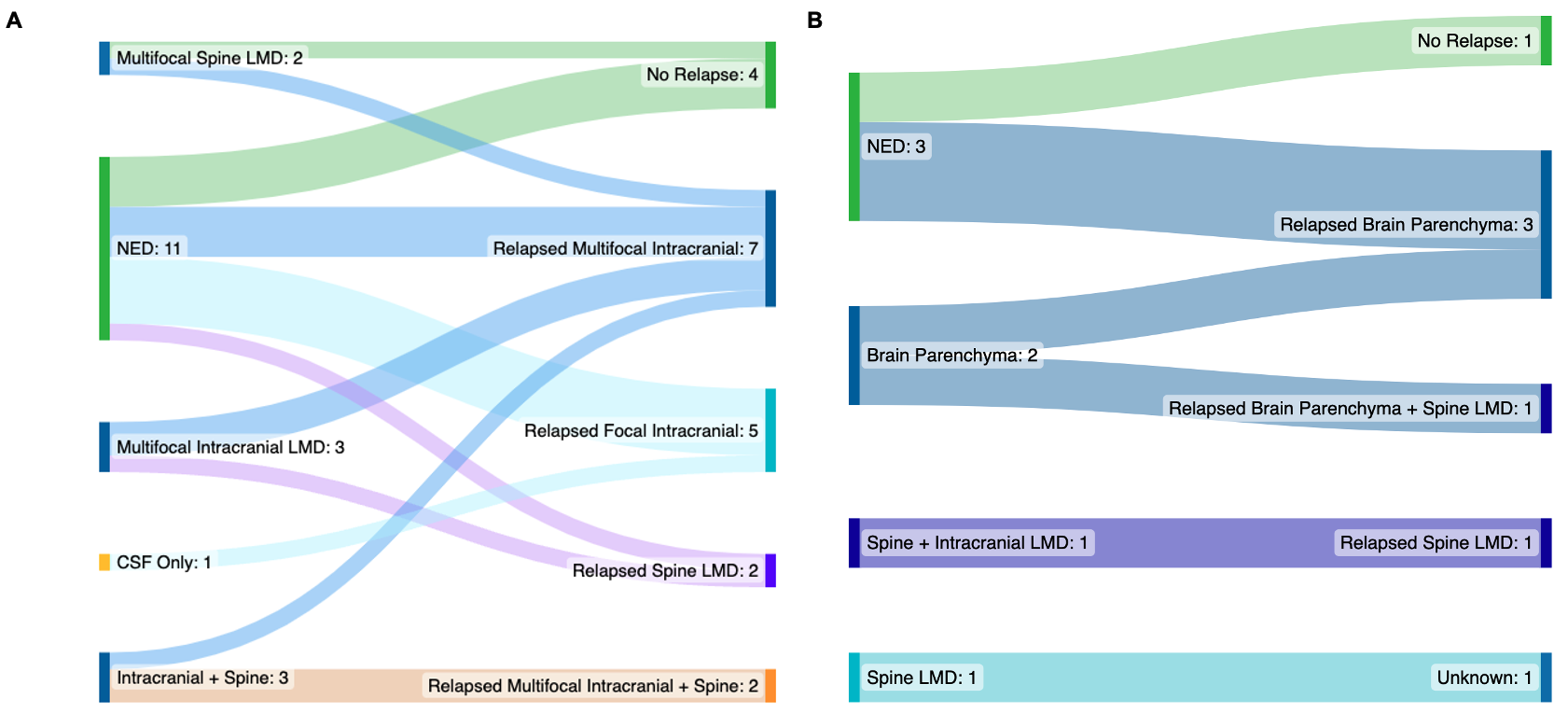
**

**Supplemental Figure 2.** Patterns of disease failure in (A) medulloblastoma and (B) ependymoma patients after cRIT. Of the 11 who were NED pre-cRIT, 8 relapsed: 4 relapsed focally intracranially (LMD n=2, ventricular n=1, parenchymal n=1), 3 relapsed multifocally intracranially (LMD n=1, LMD + intraparenchymal n=2), and 1 relapsed with spine only LMD. Of the 3 patients with multifocal intracranial disease pre-cRIT, 2 had multifocal intracranial LMD relapses, and 1 had intracranial (both LMD and intraparenchymal) as well as spine disease. Of the 2 with multifocal spine LMD, one did not relapse and one had intracranial LMD at relapse. The patient with CSF only disease at baseline had a focal intracranial relapse of LMD. Of the 3 patients with both intracranial and spine LMD, all three relapsed with multifocal LMD (intracranial alone n=1, intracranial + spine n=2).

Abbreviations: EBRT, external beam radiotherapy; LMD, leptomeningeal disease; cGy, centiGray; NED, no evidence of measurable disease; CSF, cerebrospinal fluid


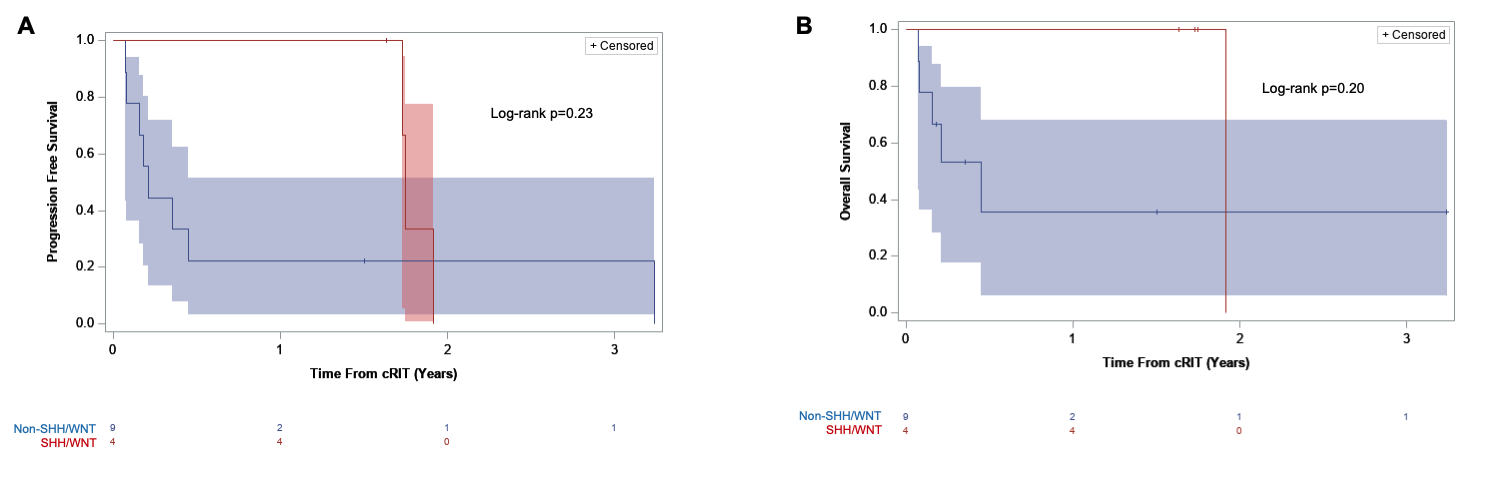


**Supplemental Figure 3.** Progression-free (A) and overall survival (B) by SHH/WNT status among the 13 medulloblastoma patients with molecular subtyping data. The median progression-free survival (PFS) among non-SHH/WNT patients was lower than SHH activated patients (0.21 years [95% CI 0.07-3.24] vs 1.74 years [95%CI 1.73-1.92]; log-rank p=0.23). Similarly, the overall survival was also lower among the non-SHH/WNT patients (0.44 years [95%CI 0.07-NR] vs 1.91 years [95%CI NR-NR]; log-rank p=0.020).

**Supplemental Table 1. Dose and Treatment Delivery of I-131-Omburtamab cRIT and External Beam Radiotherapy**

|  | **Total**  **N=27** | **Medulloblastoma**  **N=20** | **Ependymoma**  **N=7** |
| --- | --- | --- | --- |
| **cRIT** |  |  |  |
| Number of injections | 3 (1-4) | 2.5 (1-4) | 4 (2-4) |
| Total dose (mCi) | 72 (2-192) | 73 (2-192) | 72 (44-104) |
| **Radiotherapy pre-cRIT** |  |  |  |
| Number of EBRT courses* | 2 (1-5) | 2 (1-3) | 2 (1-5) |
| Time from EBRT to cRIT (yrs) | 0.9 (0.1-5.2) | 0.6 (0.1-4.9) | 1.2 (0.2-5.2) |
| **Craniospinal irradiation**** | **22 (81)** | **20 (100)** | **2 (29)** |
| Dose (cGy) | 2340 (2300-3960) | 2340 (2300-3600) | 3780 (3600-3960) |
| Total Dose with Boost (cGy) | 5400 (5040-6000) | 5400 (5400-6000) | 5490 (5040-5940) |
| Protons | 10 (37) | 10 (50) | 0 (0) |
| **Focal EBRT** | 20 (74) | 15 (71) | 5 (83) |
| Max Dose (cGy) | 3240 (1800-5940) | 3000 (1800-5000) | 5700 (4720-5940) |
| Protons | 6 (22) | 2 (10) | 4 (57) |
| **I-131-3F8** | 8 (30) | 8 (35) | 0 (0) |

Median (range), Number (%)

*The number of EBRT courses refers to the number of distinct radiation treatment plans prior to receipt of cRIT.

**The number indicates the number of individual patients who received craniospinal irradiation prior to cRIT.

Abbreviations: cRIT, compartmental radioimmunotherapy; EBRT, external beam radiotherapy

**Supplemental Table 2.** **Radiologic Findings**

**
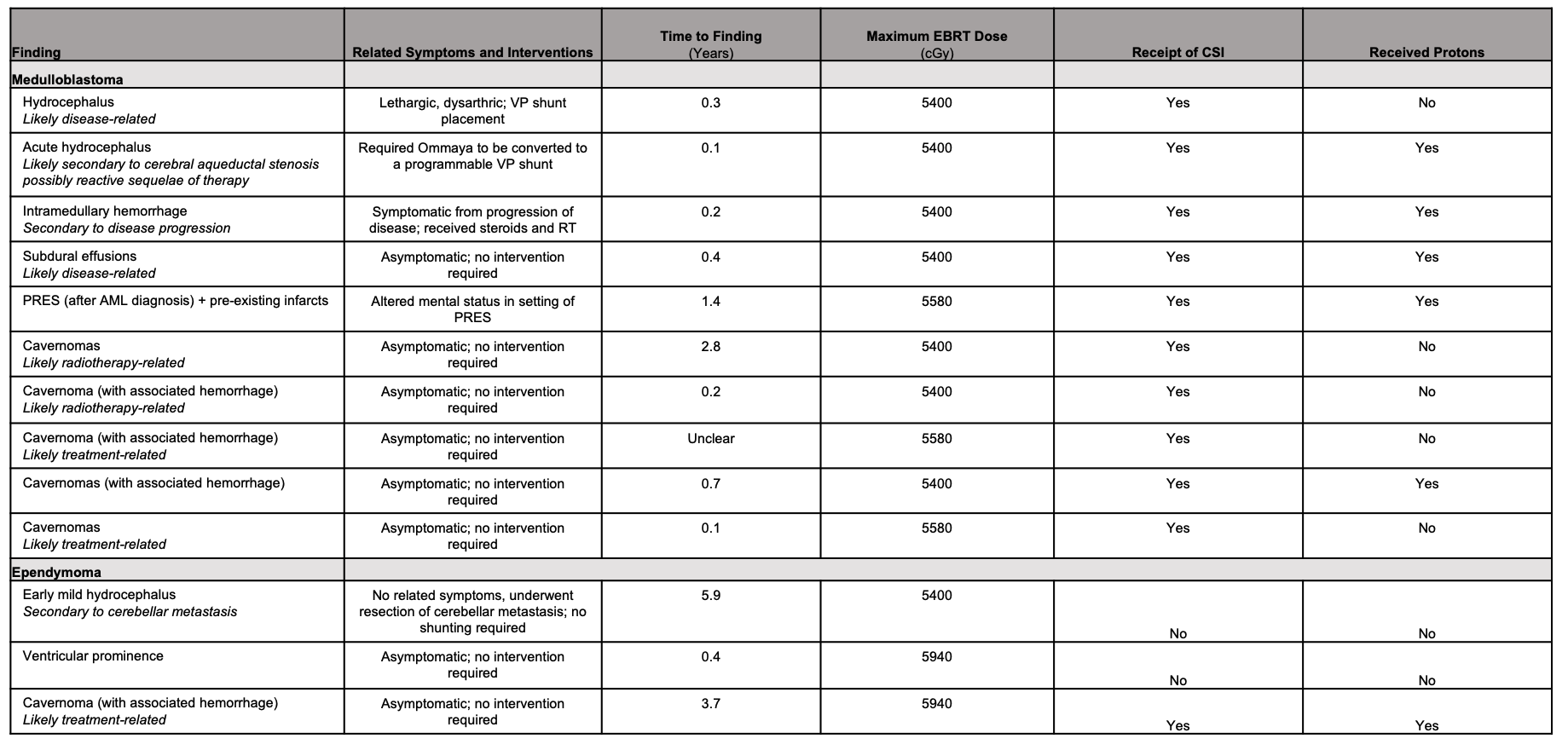
**

Each row represents a unique patient. In total, thirteen patients had radiologic events: six (46%) had cavernomas, three (23%) had hydrocephalus (one of whom was acutely symptomatic), one (8%) had ventricular prominence, one (8%) had subdural effusions, one (8%) had intramedullary hemorrhage, and one (8%) had posterior reversible encephalopathy syndrome (PRES) after developing acute myeloid leukemia.
